# Epigenome-wide exploratory study of monozygotic twins suggests differentially methylated regions to associate with hand grip strength

**DOI:** 10.1007/s10522-019-09818-1

**Published:** 2019-06-28

**Authors:** Mette Soerensen, Weilong Li, Birgit Debrabant, Marianne Nygaard, Jonas Mengel-From, Morten Frost, Kaare Christensen, Lene Christiansen, Qihua Tan

**Affiliations:** 1 Epidemiology, Biostatistics and Biodemography, Department of Public Health, University of Southern Denmark, J.B. Winsløws Vej 9B, 5000 Odense C, Denmark; 2 Department of Clinical Biochemistry and Pharmacology, Center for Individualized Medicine in Arterial Diseases, Odense University Hospital, J.B. Winsløws Vej 4, 5000 Odense C, Denmark; 3 Department of Clinical Genetics, Odense University Hospital, J.B. Winsløws Vej 4, 5000 Odense C, Denmark; 4 Endocrine Research Unit, KMEB, University of Southern Denmark, J.B. Winsløws Vej 4, 5000 Odense C, Denmark; 5 Department of Clinical Immunology, Copenhagen University Hospital, Rigshospitalet, Blegdamsvej 9, 2100 Copenhagen Ø, Denmark

**Keywords:** Epigenome-wide association study, Hand grip strength, Twin data, Longitudinal data, Pathway analyses, Comb-p

## Abstract

Hand grip strength is a measure of muscular strength and is used to study age-related loss of physical capacity. In order to explore the biological mechanisms that influence hand grip strength variation, an epigenome-wide association study (EWAS) of hand grip strength in 672 middle-aged and elderly monozygotic twins (age 55–90 years) was performed, using both individual and twin pair level analyses, the latter controlling the influence of genetic variation. Moreover, as measurements of hand grip strength performed over 8 years were available in the elderly twins (age 73–90 at intake), a longitudinal EWAS was conducted for this subsample. No genome-wide significant CpG sites or pathways were found, however two of the suggestive top CpG sites were mapped to the *COL6A1* and *CACNA1B* genes, known to be related to muscular dysfunction. By investigating genomic regions using the comb-p algorithm, several differentially methylated regions in regulatory domains were identified as significantly associated to hand grip strength, and pathway analyses of these regions revealed significant pathways related to the immune system, autoimmune disorders, including diabetes type 1 and viral myocarditis, as well as negative regulation of cell differentiation. The genes contributing to the immunological pathways were *HLA*-*B*, *HLA*-*C*, *HLA*-*DMA*, *HLA*-*DPB1*, *MYH10*, *ERAP1* and *IRF8*, while the genes implicated in the negative regulation of cell differentiation were *IRF8*, *CEBPD*, *ID2* and *BRCA1*. In conclusion, this exploratory study suggests hand grip strength to associate with differentially methylated regions enriched in immunological and cell differentiation pathways, and hence merits further investigations.

**Electronic supplementary material:**

The online version of this article (10.1007/s10522-019-09818-1) contains supplementary material, which is available to authorized users.

## Introduction

Aging is accompanied by a general loss of physical capacity that is often associated with adverse age-related outcomes and thus may have significant influences on the quality of life. For example, age-related decline in skeletal muscle strength, which is an important component of sarcopenia and frailty, is associated with physical disability, morbidity and mortality (Beaudart et al. 2017).

Hand grip strength is a simple and easily administered measurement of muscle strength that correlates well with overall muscle strength (Visser et al. 2000; Wang et al. 2005; Bohannon et al. 2012). In accordance, the average hand grip strength peaks in early adulthood, followed by a period of continuance, and then starts declining from midlife and throughout life (Frederiksen et al. 2006; Dodds et al. 2014). Hand grip strength has been associated with a number of adverse health outcomes, such as depression (van Milligen et al. 2011; Fukumori et al. 2015), cardio-metabolic risk markers and diseases (Mainous et al. 2015; Lawman et al. 2016; Lee et al. 2016), and multi-morbidity (Cheung et al. 2013; Yorke et al. 2015), as well as with an increased overall mortality risk (Newman et al. 2006; Arvandi et al. 2016). In a study of almost 140,000 individuals from 17 different countries, Leong et al. (2015) found associations between hand grip strength and incident cardiovascular disease and further confirmed that hand grip strength is a strong predictor of all-cause and in particular cardiovascular mortality. Recently, a study of 502,293 participants from the UK Biobank confirms an association to all cause mortality and cause specific mortality from cardiovascular disease, yet also puts forward associations to cause specific mortality from respiratory disease, chronic obstructive pulmonary disease and cancers, as well as to incidences of cardiovascular disease, respiratory diseases, chronic obstructive pulmonary disease and cancers (Celis-Morales et al. 2018). In addition, recent literature reviews firmly validate the associations between hand grip strength and cognitive decline (Fritz et al. 2017; Rijk et al. 2016) and functional status such as activity of daily living (Rijk et al. 2016).

Previous studies have estimated that the heritability of hand grip strength is approximately 50–60% (Frederiksen et al. 2002). However, very few consistent genetic associations have been found, and large genome-wide association studies (GWAS) of hand grip strength are scarce compared to other traits, thus leaving the molecular background of hand grip strength largely unaccounted for (Garatachea and Lucía 2013; Chan et al. 2015). The very first hand grip strength GWAS included 2088 adults (aged 55–85 years) and revealed no genome-wide significant results (Chan et al. 2015). Later, a meta-analysis of 27,581 individuals over 65 years of age from 14 different cohorts discovered one genome-wide significant association, rs752045, in an intergenic region on chromosome 8, which was replicated in 6363 individuals from three additional cohorts (Matteini et al. 2016). rs752045 is located in a DNase hypersensitivity site known to affect a binding motif of the CCAAT/enhancer-binding protein-b (CEBPB), which has been implicated in muscle cell differentiation and muscle repair (Matteini et al. 2016). Recently, an even larger multi-center GWAS of 195,180 individuals identified 16 loci including genes involved in processes such as structure and function of skeletal muscle fibers (*ACTG1*), neuronal maintenance and signal transduction (*PEX14*, *TGFA*, *SYT1*), and monogenic syndromes with involvement of psychomotor impairment (*PEX14*, *LRPPRC* and *KANSL1*) (Willems et al. 2017).

In addition to genetic studies, blood-based studies of molecular signatures may prove valuable for determining systemic factors and pathways involved in muscle strength maintenance and age-related decline. A gene expression analysis of blood messenger RNA from 698 people found the expression of the *CEBPB* gene to be significantly associated with hand grip strength as well as with physical performance (Harries et al. 2012). In a larger transcriptome-wide association study (TWAS), Pilling et al. (2016) investigated the association between hand grip strength and gene expression levels in a total of 7781 individuals aged 20–104 years from 4 independent cohorts. They identified 208 differentially expressed genes, half of which were novel in muscle-related literature, thus warranting future work on mechanisms underlying these associations (Pilling et al. 2016). Recently, Murabito et al. (2017) examined the association of 229 whole-blood microRNAs (miRNAs) with hand grip strength in a population of 5668 individuals aged 24–90 years. The study identified 93 significant miRNAs, for which the potential gene targets were involved in biological pathways of importance for muscle and aging (e.g. metabolism) (Murabito et al. 2017).

To our knowledge, only one study has compared blood-based DNA methylation with hand grip strength. In 2015, Marioni et al. performed an epigenome-wide association study (EWAS) of hand grip strength in 1085 individuals from the Lothian Birth Cohort (mean age of 69.5 years) and found no CpG sites with a *p* value < 10^−5^. However, looking at the related phenotype skeletal muscle mass, a blood-based EWAS in 1550 middle-aged women, including 50 monozygotic twin pairs discordant for hand grip strength, identified a number of significantly associated differentially methylated genomic regions, of which some showed replication in 1196 individuals: these included the *DNAH12*, *ZFP64*, *TP63* and *GUSBP11* genes, which have previously been linked to muscle differentiation and function (Livshits et al. 2016). Hence, the latter study suggests the importance of epigenetic approaches for understanding the molecular basis of muscle phenotypes such as hand grip strength, and furthermore supports the use of blood as an informative tissue in studies of muscle function.

With the aim of exploring the association between hand grip strength and DNA methylation profiles in blood cells, we studied a sample of 672 monozygotic twins with an age span of 55–90 years. The study took advantage of the twin data by applying an intra-pair approach, controlling the influence of genetic variation and lowering the influence of potential confounders such as differences in rearing environment. Furthermore, we performed a longitudinal EWAS in 192 elderly individuals (age 73–90 years at intake), for whom data on hand grip strength were available from up to five survey waves conducted over 8 years.

## Materials and methods

### Study populations

The study population comprised 672 monozygotic twins drawn from population-based, nation-wide surveys conducted within the framework of the Danish Twin Registry (Skytthe et al. 2013). Of these, 480 were participants from the Study of Middle Aged Danish Twins (MADT) and 192 were participants from the Longitudinal Study of Aging Danish Twins (LSADT) (Skytthe et al. 2013).

MADT was initiated in 1998 as a Danish nation-wide study of 4314 twins randomly selected from birth cohorts spanning 1931–1952. In 2008–2011 a follow-up study was conducted of all eligible twin pairs originally enrolled (Skytthe et al. 2013). The present study included all intact monozygotic (MZ) twin pairs from the follow-up study. LSADT is a cohort-sequential study of Danish twins aged 70 years or more, initiated in 1995. Surviving twins were followed-up every second year until 2005 (McGue and Christensen 2007). In 1997, whole blood samples were collected from 689 same-sex twins and the present study included all MZ pairs from this wave. For replication purpose we used data on 275 participants from the Study of Birth Weight Discordant Twins (BWD), a nation-wide study conducted in 2008–2010 of the most birth weight discordant twins in the twin registry (Frost et al. 2012). These samples were previously analyzed for epigenome-wide association with birth-weight discordance by Tan et al. (2014), who reported no significant findings. The demographics of the study sample are shown in Table 1.

**Table 1:** Descriptives of the study population

Sample	Survey	No. individuals	No. complete twin pairs (no. individuals)	Sex distribution [females (%)]	Mean age at blood sampling [years (SD) (range)]	Mean hand grip strength [kg (SD) (range)]	Mean height [cm (SD) (range)]
Study population (MADT + LSADT)	MADT	480	237 (474)	219 (45.6)	66.3 (6.0) (55.9–79.9)	37.2 (11.6) (15–70)	168.4 (8.8) (147–194)
	LSADT	192	84 (168)	124 (64.6)	78.5 (3.7) (73.2–90.5)	24.3 (8.3) (7–45)	165 (8.2) (145–183)
	Total	672	321 (642)	343 (51.0)	69.8 (7.8) (55.9–90.5)	33.5 (12.2) (7–70)	167.5 (8.8) (145–194)
Replication sample	BWD (young) BWD (middle-aged)	136 139	66 (132) 66 (132)	61 (44.9) 68 (48.9)	33.6 (2.0) (30.1–37.3) 63.5 (3.8) (57.4–74.1)	46.0 (12.0) (17–79) 38.5 (11.5) (14–66)	175.4 (10.2) (154–205) 170.1 (8.1) (151–188)
	Total	275	132 (264)	132 (48.0)	48.7 (15.3) (30.1–74.1)	42.2 (12.3) (14–79)	172.7 (9.5) (151–205)

As the BWD is made up by two age groups, they are listed separately here

*No.* number of, *SD* standard deviation

Informed consents were obtained from all participants and all surveys were approved by The Regional Committees on Health Research Ethics for Southern Denmark (S-VF-19980072, S-VF-20040241 and S-20090033) and were conducted in accordance with the Helsinki II declaration.

### Phenotype data

Hand grip strength was measured by a hand-held dynamometer (SMEDLEY’S dynamometer, Scandidact, Kvistgaard, Denmark), using a maximum of three measurements with the strongest hand. For LSADT the measurements were performed in 1999, while the blood sample was drawn in 1997, giving a mean age difference of 1.79 years (standard deviation (SD) = 0.28). In analysis of the LSADT data, the co-variate values (including age) were from 1997. For MADT and BWD all data were collected in the same survey wave, yet not necessarily on the same date. On average, the difference between blood sampling and measurement of hand grip strength was less than 2 weeks.

For the 192 LSADT individuals longitudinal data were available. Survey waves were performed in 1999, 2001, 2003, 2005 and 2007. In summary, 39 individuals took part in the baseline wave (1999) only, while 46, 36 and 34 individuals took part in 2, 3 or 4 waves, respectively, and 37 individuals took part in all 5 waves. The individual-level hand grip strength values decreased in the study population as a whole (see Online Resource 1, Supplementary Fig. 1), with an average decline from intake to the last measurement of − 0.49 kg/year (SD 1.42, range − 5.9; 7.3).

### DNA methylation data

DNA was isolated from buffy coat by salt precipitation, using either a manual protocol (Miller et al. 1988) or a semi-automated protocol with the Autopure System (Qiagen, Hilden, Germany). Bisulfite conversion of 500 ng genomic DNA was performed using the EZ Methylation Gold kit (Zymo Research, Orange County, CA, USA) and DNA methylation was measured using the Infinium HumanMethylation450K BeadChip (Illumina, San Diego, CA, USA) at Leiden University Medical Center or GenomeScan B.V., Leiden, The Netherlands, according to the manufacturer’s protocols. The BeadChip images were scanned using the iScan system.

The DNA methylation data were prepared on four different occasions giving four datasets; the BWD dataset (N = 296), the MADT dataset (N = 498) and two separate LSADT datasets (N = 224 and N = 86, respectively). Quality control and normalization were performed similarly to Tobi et al. 2015 and separately for each dataset. Data pre-processing was done using the free R packages *MethylAid* (van Iterson et al. 2014) and *minfi* (Aryee et al. 2014). Samples were excluded, first if less than 95% of the probes had a detection P-value < 0.01, or second if the samples failed based on the internal quality control probes used by *MethylAid* (van Iterson et al. 2014). The sex of the individuals was confirmed by considering the values of the X chromosome probes by multidimensional scaling. The DNA methylation data were annotated using GRCh37/hg19. In total, 4 BWD twin pairs and 6 MADT twin pairs were excluded due to poor sample quality (8) or sex discrepancy (2). Probes were excluded if they had a detection P-value > 0.01, a raw intensity value of zero, a low bead count (< 3 beads), a measurement success rate below 95% or were identified as being cross-reactive (Chen et al. 2013). In total, between 32,055 and 33,316 CpGs out of the 485,512 CpGs on the array were excluded for of the four datasets. After probe filtering, a sample success rate of 0.95 was applied; no samples were excluded. Normalization was carried out by Functional normalization (Fortin et al. 2014) with four principle components (PCs) for the MADT and LSADT datasets and 5 PCs for the BWD dataset. The present study includes CpGs overlapping between the MADT and LSADT datasets (452,178 CpGs). Before statistical analysis, the β-values were transformed to M-values by logit transformation, presenting a distribution more suitable for statistical testing (Du et al. 2010).

Blood leukocyte subtypes (monocytes, lymphocytes, basophils, neutrophils and eosinophils) counted using a Coulter LH 750 Haematology Analyser were available for 483 of the 492 MADT individuals and for 212 of the 292 BWD individuals. For the remaining individuals the blood counts were imputed by a modified version of the *PredictCellComposition* method (www.github.com/mvaniterson/predictcellcomposition), employing partial least squares regression after setting values of 0 to 0.0001, i.e. inflating percentages below the minimum value observed (0.0099), and transforming the cell composition using an isometric log ratio transformation.

### Statistical analysis

#### Cross-sectional analyses

A principle component analysis (PCA) of the methylation data found the top 4 PCs to describe the majority of the variance in the data, as the subsequent PCs accounted for less than 3% of the variance (data not shown). Still, as PC1 correlated highly (correlation coefficient: 0.99) with sex, only PC2–4 were included in the statistical models. Furthermore, as the technical co-variates DNA plate number, 450K array number and dataset reflected much of the same variance, only the dataset co-variate was included. Cell counts for monocytes, lymphocytes and eosinophils were included in all statistical models, while basophil counts were excluded due to low variability, and neutrophil cell counts were excluded as neutrophil and lymphocyte cell counts reflected much of the same variance. Finally, age and height were included in all statistical models, as they associate with hand grip strength (Frederiksen et al. 2006), which was also found for the present data (data not shown).

Cross-sectional EWAS with single CpG methylation levels as the outcome and hand grip strength as the explanatory variable, were performed with the use of linear regression models, first on an individual level (individual level EWAS) and next on a twin pair level using an intra-pair design (twin pair level EWAS).

The individual level analysis was carried out with a mixed effect model using twin pairing as a random factor to accommodate for correlations between twins in a pair. Sex, age at blood sampling, height, cell counts (monocytes, lymphocytes and eosinophils) and PC2–4 were included as fixed effects and a dataset variable as a further random effect.

In the twin pair level analysis, a linear regression model was applied (similarly to Starnawska et al. 2017) to test the association between intra-pair DNA methylation difference and intra-pair difference in hand grip strength, while adjusting for sex, mean age of the twin pair at time of blood sampling, as well as for the intra-pair difference in cell counts (monocytes, lymphocytes and eosinophils), height and PCs 2–4. For the 321 intact twin pairs of the present study population the average hand grip strength was 34.0 kg (SD 12.2, range 7–70), while the average intra-pair difference was 4.4 kg (SD 3.8, range 0–21). The intra-pair difference for a given co-variate was calculated by subtracting the co-variate value of the twin with the lower hand grip strength from the co-variate value of the co-twin with the higher hand grip strength. In a sub-analysis, the twin pair level model was applied to the 50% most discordant twin pairs with respect to hand grip strength, thus removing twin pairs with no or little intra-pair difference.

The replication analysis in the 275 BWD individuals was performed as described above, with additional adjustment for birth weight or intra-pair difference in birth weight.

All statistical analyses were carried out in R version 3.3.1.

#### Longitudinal analyses

An EWAS of hand grip strength over time was carried out by a growth curve model, which employs a linear mixed-effects model with hand grip strength at the different time points as the outcome and the single CpG methylation level, time from baseline to survey, sex, age at blood sampling, cell counts (monocytes, lymphocytes and eosinophils), height and PCs 2–4 as fixed effects. A dataset variable, a twin pairing variable and a twin ID variable (allows individual varying baseline levels) were included as random effects, whereby the twin ID specific effect was nested within the twin pair specific effect. This initial random intercept model was extended by a random slope model (allows individual varying changes in hand grip strength over time). Considering that the two models gave very similar results, although slightly higher p-values in the random slope model (data not shown), only the results from the random intercept model are reported. All statistical analyses were carried out in R version 3.3.1.

#### Pathways analyses of EWAS findings

CpGs with an uncorrected p-value < 10^−3^ were annotated using the information by Illumina and the corresponding genes were used for overrepresentation enrichment analysis (ORA) against the genes on the 450K array using the Kyoto Encyclopedia of Genes and Genomes (KEGG) and Reactome databases available through the WEB-based GEne SeT AnaLysis Toolkit (WebGestalt) (Wang et al. 2017). Moreover, an ORA sub-analysis restricted to the 134,070 CpG sites annotated to the Function Feature Type TSS200 (transcription start site (TSS) to − 200 nt upstream of TSS) or TSS1500 (− 200 to − 1500 nt upstream of TSS) was performed. The number of genes investigated and annotated to the databases can be seen in the Online Resource 1, Supplementary Table 1.

#### Differentially methylated regions

In order to identify differentially methylated regions (DMR), the comb-p approach was applied (Pedersen et al. 2012) for the results obtained in the cross-sectional and longitudinal EWAS. The comb-p has been reported to have high control of false-positive rate and the best sensitivity compared to other popular DMR tools, such as bumphunter, DMRcate and probe lasso (Mallik et al. 2018). The following settings were used: p-value was set to 0.1 to seed a region; maximum 500 bps to skip before finding next seed, if new seed was not found, the region is ended. Gene regions identified were annotated using the BioMart (www.biomart.org) and Ensemble (www.ensembl.org) databases. The gene regions passing correction for multiple testing by false discovery rate (FDR) (see below) were investigated by MSigDB genomic regions enrichment analysis (GREA) by the Genomic Regions Enrichment of Annotations Tools (GREAT) (http://great.stanford.edu) using the BED file output from comb-p as input. The hypergeometric test over genes was applied.

#### Adjustment for multiple testing

In all analyses adjustment for multiple testing was performed by the Benjamini–Hochberg FDR correction method (Benjamini and Hochberg 1995). The suggested genome-wide significance threshold for EWAS analyses using 450K data has been proposed to be p < 10^−6^ (Tsai and Bell 2015). However, as we consider this an exploratory study, and in order to give an overview of the top results, we report all findings with an unadjusted p < 10^−5^ for the EWAS results.

## Results

The demographics of the study sample are shown in Table 1. All Supplementary results can be found in the Online Resource 1.

### Single CpG epigenome-wide association studies of hand grip strength

Epigenome-wide association studies (EWAS) were performed both on the intake hand grip values for the entire study population, as well as on longitudinal hand grip measurements, which were available for the elderly twin sample (LSADT). The qq-plots of the analyses are shown in the Online Resource 1, Supplementary Fig. 2, while the EWAS results are listed in the Online Resource 1, Supplementary Tables 2–5.

No genome-wide significant associations were observed in the cross-sectional analyses of the 672 individuals (see Table 2A–C). Five CpG sites displayed a p-value below the cut-off of 1 × 10^−5^ in the individual level EWAS: four of these were located in gene regions, i.e. in the d-glutamate cyclase (*DGLUCY*), NLR family CARD domain containing (*NLRC5*), tec protein tyrosine kinase (*TEC*) and iroquois homeobox 4 (*IRX4*) genes, respectively, and 1 CpG site was in an intragenic region, the nearest gene being MT-RNR2 Like 1 (*MTRNR2L1*). Similarly, the twin pair level EWAS displayed 5 CpG sites with a p-value < 1 × 10^−5^: four were located in gene regions, i.e. in the solute carrier organic anion transporter family member 3A1 (*SLCO3A1*), calcium voltage-gated channel subunit alpha1 (*CACNA1B*), RNA binding motif protein 33 (*RBM33*) and LIM domain binding 2 (*LDB2*) genes, respectively and 1 CpG site was in an intragenic region, the nearest gene being Lengsin (*LGSN*). The CpG site cg01763947 in *SLCO3A1* was also found to be associated in the analysis of the 50% most discordant twin pairs, together with 6 additional CpG sites.

**Table 2:** Overview of the results (p-value < 10^−5^) from the (A–C) cross-sectional EWAS and the (D) longitudinal EWAS (LSADT sample) of hand grip strength

	CpG	Estimate	SE	P-value	Chr	Position (bp)	Gene	Distance to gene (bp)	Gene—CGI feature
(A) Individual level analysis	cg14304073	− 0.0029	0.0006	2.53E−06	14	91,691,190	*DGLUCY*	0	3′UTR—open sea
	cg16411857	0.0068	0.0015	5.87E−06	16	57,023,191	*NLRC5*	0	TSS1500—island
	cg14057711	− 0.0083	0.0018	6.71E−06	17	22,203,489	*MTRNR2L1*	− 179,498	IGR—open sea
	cg13469425	0.0113	0.0025	7.39E−06	4	48,175,353	*TEC*	0	Body—open sea
	cg03398865	− 0.0058	0.0013	9.20E−06	5	1,879,554	*IRX4*	0	Body—shore
(B) Intra-pair analysis all	cg01763947	0.0160	0.0032	1.09E−06	15	92,459,894	*SLCO3A1*	0	Body—shore
	cg11680027	0.0123	0.0026	2.76E−06	6	63,921,394	*LGSN*	64,462	IGR—open sea
	cg14140691	0.0100	0.0022	4.97E−06	9	141,012,312	*CACNA1B*	0	Body—shelf
	cg18809570	0.0116	0.0025	5.25E−06	7	155,437,147	*RBM33*	0	TSS200—island
	cg14187877	0.0253	0.0055	5.75E−06	4	16,679,554	*LDB2*	0	Body—open sea
(C) Intra-pair analysis top 50% discordant	cg01763947	0.0274	0.0055	1.47E−06	15	92,459,894	*SLCO3A1*	0	Body—shore
	cg06663068	0.0176	0.0036	2.11E−06	3	9,994,061	PRRT3; PRRT3*	0	1st Exon; 5′UTR—island
	cg15549075	0.0140	0.0029	2.77E−06	2	46,098,874	*PRKCE*	0	Body—open sea
	cg27653831	0.0318	0.0067	4.26E−06	16	67,700,515	C16orf86;ENKD1; ENKD1*	0	TSS1500; 5′UTR; 1stExon—island
	cg01874084	0.0314	0.0066	4.86E−06	12	56,320,851	*WIBG*	0	Body—island
	cg05870108	− 0.0466	0.0100	6.80E−06	X	106,749,745	*FRMPD3*-*AS1*	6468	IGR—shore
	cg10314909	− 0.0278	0.0060	7.46E−06	1	2,524,890	*MMEL1*	0	Body—shelf
(D) Longitudinal analysis (LSADT sample)	cg15245520	2.2127	0.4781	5.00E−06	21	47,411,200	*COL6A1*	0	Body—shelf
	cg04927341	1.4535	0.3190	6.96E−06	1	86,576,497	*COL24A1*	0	Body
	cg09714181	− 0.6239	0.1371	7.20E−06	5	126,853,350	*PRRC1*	0	5′UTR;1stExon—island
	cg20569588	− 1.5613	0.3448	7.79E−06	1	55,267,285	*TTC22*	0	TSS1500;TSS1500—shore
	cg17070108	2.1001	0.4649	8.22E−06	12	8,758,006	*AICDA*	0	Body—island

*SE* standard error, *Chr* chromosome, *bp* base pairs, *CGI* CpG island, *UTR* untranslated region, *TSS* transcription start site, *IGR* intergenic region (CpG is assigned to nearest gene)

*CpG holds different gene feature depending on transcript. Annotation is based on Build 37

None of the 16 top-ranked CpG sites listed in Table 2A–C replicated convincingly in the BWD twins (see the Online Resource 1, Supplementary Table 6).

The longitudinal analysis of hand grip strength in the LSADT sample revealed 5 CpG sites in 5 different genes with a p-value below the cut-off of 1 × 10^−5^: the collagen type VI alpha 1 chain (*COL6A1*), collagen type XXIV alpha 1 chain (*COL24A1*), proline rich coiled-coil 1 (*PRRC1*), tetratricopeptide repeat domain 22 (*TTC22*) and activation induced cytidine deaminase (*AICDA*) genes, respectively (see Table 2D).

In the overrepresentation enrichment analyses (ORA) against the KEGG and Reactome databases of the CpG sites with an uncorrected p-value below 1 × 10^−3^, none of the identified pathways reached statistical significance after correction for multiple testing. All ORA results with an uncorrected p-value < 0.05 are summarized in the Online Resource 1, Supplementary Table 7.

### Analysis of differentially methylated regions

The EWAS results obtained were used for identification of differentially methylation regions (DMRs) by the comb-p algorithm. In total, 70, 58, 51 and 63 DMRs displayed a FDR p-value below 0.05 for the individual level EWAS, the twin pair level EWAS, the twin pair level EWAS for the 50% most discordant pairs and the longitudinal EWAS, respectively (see Tables 3, 4, 5 and 6). Of these, 60, 49, 41 and 54 DMRs, respectively, were annotated to gene regions, while the remaining DMRs were annotated to intergenic regions.

**Table 3:** DMRs associated to hand grip strength (FDR < 0.05) in the individual level EWAS

DMR position	Lowest p-value	Length	Region p-value	FDR p-value	Gene name/nearest gene (distance in bp)
chr8:48675647–48676093	2.61E−11	7	2.61E−11	2.58E−08	*PRKDC* (− 10022)
chr17:37024020–37024761	1.01E−09	6	1.15E−09	6.85E−07	*LASP1* (− 2092)
chr4:5021084–5021695	4.55E−10	9	6.60E−09	4.77E−06	*CYTL1*
chr6:32223076–32223431	1.39E−08	10	1.39E−08	1.73E−05	*TSBP1*-*AS1* (− 412)
chr6:33040535–33041584	1.69E−10	11	5.63E−08	2.37E−05	*HLA*-*DPA1*
chr6:32920102–32921234	1.89E−08	12	5.79E−08	2.26E−05	*AL645941.2*
chr6:32920102–32921234	1.89E−08	12	5.79E−08	2.26E−05	*HLA*-*DMA*
chr1:223566127–223567174	4.65E−07	11	1.65E−07	6.94E−05	*C1orf65*
chr6:32120203–32121556	4.03E−10	35	2.43E−07	7.95E−05	*PRRT1*
chr6:32120203–32121556	4.03E−10	35	2.43E−07	7.95E−05	*PPT2*
chr5:96078430–96079434	5.05E−06	5	3.31E−07	1.46E−04	*CAST*
chr1:16163479–16164123	7.29E−08	7	3.49E−07	2.40E−04	*FLJ37453*
chr6:27482641–27483070	3.69E−07	4	3.70E−07	3.80E−04	*AL021918.1*
chr6:28972937–28973829	1.94E−08	15	4.85E−07	2.40E−04	*ZNF311*
chr7:2801424–2802977	4.47E−08	11	1.06E−06	3.03E−04	*AMZ1*
chr7:2801424–2802977	4.47E−08	11	1.06E−06	3.03E−04	*GNA12*
chr3:42977777–42978249	3.03E−07	8	1.44E−06	1.35E−03	*AC092042.3*
chr3:42977777–42978249	3.03E−07	8	1.44E−06	1.35E−03	*KRBOX1*
chr3:42977777–42978249	3.03E−07	8	1.44E−06	1.35E−03	*KRBOX1*-*AS1*
chr4:46126066–46126374	2.85E−06	6	2.85E−06	4.07E−03	*GABRG1*
chr2:731073–731595	3.54E−06	6	3.65E−06	3.09E−03	*AC092159.1*
chr5:177412586–177413022	4.73E−06	5	4.73E−06	4.78E−03	*AC106795.*3 (− 2967)
chr7:156400033–156400991	3.38E−06	7	4.93E−06	2.27E−03	*LINC01006*
chr2:8596908–8597924	1.13E−04	6	5.15E−06	2.24E−03	*LINC00299* (+ 74173)
chr21:47604052–47605175	3.68E−05	8	5.52E−06	2.17E−03	*AP001468.1*
chr21:47604052–47605175	3.68E−05	8	5.52E−06	2.17E−03	*SPATC1L*
chr6:33039396–33039805	5.77E−06	7	5.77E−06	6.22E−03	*HLA*-*DPA1*
chr19:12831659–12832213	5.73E−06	4	5.90E−06	4.69E−03	*TNPO2*
chr16:83171068–83171315	6.35E−06	4	6.35E−06	1.13E−02	*CDH13*
chr6:31323397–31323857	6.54E−06	5	6.77E−06	6.49E−03	*HLA*-*B*
chr16:85878940–85879146	6.81E−06	4	6.81E−06	1.45E−02	*AC018695.1* (+ 29004)
chr1:202172535–202172913	8.84E−06	5	8.84E−06	1.03E−02	*LGR6*
chr19:12983876–12984646	1.54E−04	3	9.53E−06	5.45E−03	*MAST1*
chr1:117529478–117530010	9.46E−06	5	9.81E−06	8.11E−03	*PTGFRN*
chr1:870791–872236	4.39E−06	10	1.14E−05	3.48E−03	*SAMD11*
chr1:109204168–109204822	5.39E−05	6	1.18E−05	7.94E−03	*HENMT1* (+ 674)
chr20:42142224–42143490	5.30E−06	27	1.25E−05	4.35E−03	*L3MBTL1*
chr6:159238463–159238745	1.31E−05	3	1.31E−05	2.03E−02	*EZR*
chr7:16890431–16891080	1.04E−05	6	1.46E−05	9.89E−03	*AC073333.2* (+ 731)
chr6:31431312–31431903	4.83E−05	5	1.52E−05	1.13E−02	*HCP5*
chr6:149772150–149772893	4.66E−05	6	1.54E−05	9.11E−03	*ZC3H12D*
chr1:161008127–161008978	6.03E−06	11	1.58E−05	8.17E−03	*AL591806.3*
chr1:161008127–161008978	6.03E−06	11	1.58E−05	8.17E−03	*TSTD1*
chr10:102278918–102280552	1.89E−05	13	1.62E−05	4.38E−03	*SEC31B*
chr10:102278918–102280552	1.89E−05	13	1.62E−05	4.38E−03	*AL133352.1*
chr10:102278918–102280552	1.89E−05	13	1.62E−05	4.38E−03	*NDUFB8*
chr17:8378756–8379226	3.07E−06	5	1.64E−05	1.53E−02	*NDEL1*
chr17:8378756–8379226	3.07E−06	5	1.64E−05	1.53E−02	*MYH10*
chr15:50474069–50474622	9.07E−06	9	2.33E−05	1.85E−02	*ATP8B4*
chr15:50474069–50474622	9.07E−06	9	2.33E−05	1.85E−02	*SLC27A2*
chr7:1893710–1894689	1.57E−04	7	2.81E−05	1.26E−02	*MAD1L1*
chr1:3806715–3808170	9.38E−05	7	2.97E−05	8.98E−03	*C1orf174*
chr3:21792248–21792992	6.99E−06	8	4.26E−05	2.50E−02	*ZNF385D*
chr1:156260918–156261404	1.72E−05	6	4.64E−05	4.13E−02	*TMEM79*
chr1:156260918–156261404	1.72E−05	6	4.64E−05	4.13E−02	*C1orf85*
chr2:75425832–75426299	1.50E−05	3	4.80E−05	4.44E−02	*TACR1*
chr1:202130344–202131185	1.03E−04	5	4.90E−05	2.54E−02	*PTPN7*
chr5:321681–322817	1.07E−03	6	5.21E−05	2.01E−02	*PDCD6*
chr5:321681–322817	1.07E−03	6	5.21E−05	2.01E−02	*AHRR*
chr6:28828946–28829765	1.57E−05	19	5.81E−05	3.09E−02	*LINC01623*
chr6:28828946–28829765	1.57E−05	19	5.81E−05	3.09E−02	*RPL13P*
chr19:55549414–55550251	3.34E−05	9	6.19E−05	3.21E−02	*GP6*
chr19:55549414–55550251	3.34E−05	9	6.19E−05	3.21E−02	*AC011476.3*
chr19:55549414–55550251	3.34E−05	9	6.19E−05	3.21E−02	*AC011476.2*
chr17:41277847–41278713	4.05E−05	15	6.22E−05	3.12E−02	*NBR2*
chr17:1944831–1945955	5.84E−05	9	9.94E−05	3.83E−02	*DPH1*
chr17:1944831–1945955	5.84E−05	9	9.94E−05	3.83E−02	*OVCA2*
chr19:58569986–58570996	5.42E−05	10	1.09E−04	4.67E−02	*ZNF135*
chr6:41375881–41377289	5.66E−05	6	1.41E−04	4.33E−02	*FOXP4*-*AS1* (− 86710)
chr12:115130855–115132762	3.38E−03	13	1.63E−04	3.71E−02	*TBX3* (+ 10793)

*FDR* false discovery rate corrected p-value, *bp* base pairs

**Table 4:** DMRs associated to hand grip strength (FDR < 0.05) in the twin pair level EWAS

DMR position	Lowest p-value	Length	Region p-value	FDR p-value	Gene name/nearest gene (distance in bp)
chr6:28972891–28973829	2.02E−17	16	2.04E−12	9.60E−10	*ZNF311*
chr3:182816738–182817627	6.16E−11	13	8.21E−11	4.08E−08	*MCCC1*
chr8:125462982–125463683	2.28E−09	7	2.38E−09	1.50E−06	*TRMT12*
chr14:24422368–24423200	2.80E−10	9	1.05E−08	5.56E−06	*DHRS4*-*AS1*
chr14:24422368–24423200	2.80E−10	9	1.05E−08	5.56E−06	*DHRS4*
chr6:33040535–33041584	5.23E−09	11	2.87E−07	1.21E−04	*HLA*-*DPA1*
chr19:55972504–55973339	4.14E−08	10	4.52E−07	2.39E−04	*ISOC2*
chr3:5137549–5138171	6.81E−08	5	6.47E−07	4.60E−04	*AC090955.1* (− 3763)
chr2:8597159–8597924	3.98E−04	5	1.48E−06	8.53E−04	*LINC00299* (+ 74173)
chr1:24229232–24229683	1.96E−06	5	1.96E−06	1.92E−03	*CNR2*
chr2:47382287–47382740	2.31E−06	7	2.31E−06	2.25E−03	*C2orf61*
chr2:47382287–47382740	2.31E−06	7	2.31E−06	2.25E−03	*AC073283.3*
chr6:30881112–30882214	5.09E−09	31	2.39E−06	9.57E−04	*GTF2H4*
chr6:30881112–30882214	5.09E−09	31	2.39E−06	9.57E−04	*VARS2*
chr12:49937852–49938019	2.83E−06	3	2.83E−06	7.46E−03	*KCNH3*
chr6:33039396–33039805	3.14E−06	7	3.14E−06	3.38E−03	*HLA*-*DPA1*
chr18:60051870–60052465	3.45E−06	5	3.84E−06	2.85E−03	*TNFRSF11A*
chr6:170589483–170589898	4.44E−06	4	4.44E−06	4.71E−03	*LINC01624* (+ 923)
chr5:138211043–138211185	5.19E−06	6	5.19E−06	1.60E−02	*CTNNA1*
chr5:138211043–138211185	5.19E−06	6	5.19E−06	1.60E−02	*LRRTM2*
chr21:47604052–47605175	2.36E−04	8	7.00E−06	2.75E−03	*AP001468.1*
chr21:47604052–47605175	2.36E−04	8	7.00E−06	2.75E−03	*SPATC1L*
chr1:1564422–1565028	1.45E−04	6	7.68E−06	5.58E−03	*MIB2*
chr15:98195808–98196334	7.46E−06	6	7.69E−06	6.44E−03	*LINC00923* (− 89640)
chr2:165811816–165812298	7.39E−06	6	7.75E−06	7.08E−03	*SLC38A11*
chr7:27182493–27184031	4.51E−06	23	9.47E−06	2.72E−03	*AC004080.6*
chr7:27182493–27184031	4.51E−06	23	9.47E−06	2.72E−03	*HOXA*-*AS3*
chr7:27182493–27184031	4.51E−06	23	9.47E−06	2.72E−03	*HOXA3*
chr7:27182493–27184031	4.51E−06	23	9.47E−06	2.72E−03	*HOXA5*
chr12:7315351–7315566	1.02E−05	4	1.02E−05	2.07E−02	*AC018653.*1 (− 3919)
chr12:54446019–54447350	2.28E−06	18	1.24E−05	4.12E−03	*HOXC4*
chr7:27231204–27232151	1.02E−04	5	1.67E−05	7.74E−03	*AC004080.2*
chr9:133306495–133306792	1.98E−05	2	1.98E−05	2.90E−02	*HMCN2*
chr2:3704363–3705179	2.23E−05	8	2.29E−05	1.23E−02	*ALLC* (− 1422)
chr7:130131480–130132791	3.38E−06	33	2.37E−05	7.97E−03	*MEST*
chr22:46449430–46450115	2.22E−05	11	2.56E−05	1.64E−02	*AL121672.3*
chr22:46449430–46450115	2.22E−05	11	2.56E−05	1.64E−02	*PRR34*-*AS1*
chr22:46449430–46450115	2.22E−05	11	2.56E−05	1.64E−02	*C22orf26*
chr22:46449430–46450115	2.22E−05	11	2.56E−05	1.64E−02	*MIRLET7BHG*
chr5:134879326–134880437	1.98E−04	6	2.74E−05	1.08E−02	*NEUROG1* (+ 8798)
chr2:121776314–121776605	3.25E−05	3	3.25E−05	4.81E−02	*GLI2* (+ 26376)
chr10:14372431–14372914	3.79E−05	5	3.97E−05	3.57E−02	*FRMD4A*
chr17:6899085–6899889	6.27E−06	14	4.10E−05	2.23E−02	*ALOX12*-*AS1*
chr17:6899085–6899889	6.27E−06	14	4.10E−05	2.23E−02	*ALOX12*
chr17:6899085–6899889	6.27E−06	14	4.10E−05	2.23E−02	*AC040977.2*
chr19:45737208–45738116	2.59E−05	10	4.20E−05	2.02E−02	*MARK4*
chr19:45737208–45738116	2.59E−05	10	4.20E−05	2.02E−02	*EXOC3L2*
chr7:56515510–56516505	2.14E−06	12	4.33E−05	1.91E−02	*AC092447.7*
chr1:223566278–223567174	3.24E−06	10	4.80E−05	2.34E−02	*C1orf65*
chr6:42927940–42928921	7.20E−06	27	4.84E−05	2.16E−02	*GNMT*
chr11:61594965–61596756	1.79E−03	12	5.83E−05	1.43E−02	*FADS2*
chr11:61594965–61596756	1.79E−03	12	5.83E−05	1.43E−02	*FADS1*
chr1:64668576–64669600	1.02E−04	12	6.45E−05	2.74E−02	*UBE2U*
chr5:118603742–118604834	1.75E−04	10	7.03E−05	2.80E−02	*TNFAIP8*
chr6:32976805–32977675	3.32E−05	12	7.45E−05	3.72E−02	*HLA*-*DOA*
chr6:30069659–30070404	5.78E−06	17	8.13E−05	4.71E−02	*TRIM31* (− 1015)
chr5:140535392–140536497	4.73E−06	9	9.87E−05	3.87E−02	*PCDHB17P*
chr1:18967020–18968001	9.30E−05	6	1.04E−04	4.56E−02	*PAX7*

*FDR* false discovery rate corrected p-value, *bp* base pairs

Applying the comb-p output for functional enrichment analysis using the GREAT tool (http://great.stanford.edu), revealed 8 significant pathways for the individual level EWAS; of these pathways, 7 were related to immune function or (auto)immune diseases, while 1 was related to C-MYC transcriptional repression (see Table 7). No pathways passed correction for multiple testing for the twin pair EWAS, the twin pair level EWAS for the 50% most discordant pairs and the longitudinal EWAS. All GREAT results with an uncorrected p-value < 0.05 are summarized in the Online Resource 1, Supplementary Table 8.

## Discussion

In this study we explored the association between variation in the epigenome and physical functioning represented by hand grip strength using both cross-sectional and longitudinal measures of hand grip strength. The use of twins enabled us to explore epigenetic association independent of the genetic background of the individuals.

Although we did not identify any epigenome-wide significant associations of single CpG sites, some biologically plausible genes were observed (Table 2): the top CpG site of the longitudinal EWAS was located in *COL6A1* encoding the alpha 1 subunit of type VI collagen. Collagens are extracellular matrix proteins, maintaining the integrity of various tissues. Mutations in *COL6A1* have been reported to cause, among others, Bethlem myopathy (omim.org/entry/158810) and Ullrich Congenital Muscular Dystrophy 1 (omim.org/entry/254090) characterized by increased skeletal muscle weakness and joint stiffness in infancy. Furthermore, a top CpGs of the twin pair EWAS was located in the calcium voltage-gated channel subunit alpha1B (*CACNA1B*) gene, encoding the alpha-1B pore-forming subunit of an N-type voltage-dependent calcium channel. Voltage-dependent calcium channels are involved in calcium-dependent processes, including muscle contraction, hormone or neurotransmitter release, gene expression and cell death. Mutations in *CACNA1B* have been reported for, among others, dystonia 23, characterized by involuntary muscle contraction (omim.org/entry/614860). Both genes have been reported to be expressed across a broad range of tissues, including whole blood and skeletal muscle (www.genecards.org).

Only one study on the epigenetics of hand grip strength has been published so far (Marioni et al. 2015) with no significant CpG sites or pathways reported. However, 3 GWAS (Chan et al. 2015, Matteini et al. 2016 and Willems et al. 2017), 2 TWAS (Harries et al. 2012 and Pilling et al. 2016) and 1 genome-wide miRNA study (Murabito et al. 2017) have explored hand grip strength. None of these studies report overlapping genes among their top findings, and none of the suggestive genes of the present study were reported in these studies, neither among the top findings nor among the nominal significant genes reported by Harries et al. (2012), Chan et al. (2015), and Matteini et al. (2016). Furthermore, no significant pathways were identified in the ORA of the present study; 4 pathways did relate to muscle contraction, yet to cardiac and vascular smooth (blood vessel) muscle contraction, not skeletal muscle function (Online Resource 1, Supplementary Table 7). Of the previous studies, a TWAS (Pilling et al. 2016), a genome-wide miRNA study (Murabito et al. 2017) and a GWAS (Willems et al. 2017) reported pathway analyses. Due to differences in methodology a complete comparison was not possible, nevertheless, if grouping pathways by the overall hierarchical level of functional events as defined by the databases, some overlap between the present ORA and the previous studies was seen: pathways related to gene expression (Pilling et al. 2016), signal transduction, the neuronal system and cell cycle/death (Murabito et al. 2017), the immune system (Pilling et al. 2016 and Murabito et al. 2017), DNA repair (Willems et al. 2017), RNA metabolism (Murabito et al. 2017) and protein metabolism (Pilling et al. 2016, Murabito et al. 2017 and Willems et al. 2017). Such overlap could indicate that these processes might relate to hand grip strength, although it must be stressed that these overall categories are broad and hence can reflect a rather broad array of biological functions.

Contrary to the single CpG analysis, the analysis of differentially methylated regions (DMRs), with enriched power by combining multiple CpGs, did show significant findings; of the 203 unique gene regions identified (Tables 3, 4, 5, 6), 5 have been reported by previous studies on hand grip strength: the PLEKHB1 pleckstrin homology domain containing B1 (*PLEKHB1*) and *O*-6-methylguanine-DNA methyltransferase (*MGMT*) genes in the GWAS of Matteini et al. (2016) and Willems et al. (2017), respectively, and the tachykinin receptor 1 (*TACR1*), G protein-coupled receptor 19 (*GPR19*) and prostaglandin F2 receptor inhibitor (*PTGFRN*) genes in the TWAS by Harries et al. (2012). Interestingly, mutations in *PLEKHB1* have been reported to cause amyotrophic lateral sclerosis, characterized by problems with muscle control and movement (omim.org/entry/105400).

**Table 5:** DMRs associated to hand grip strength (FDR < 0.05) in the twin pair level EWAS the 50% most discordant pairs

DMR position	Lowest p-value	Length	Region p-value	FDR p-value	Gene name/nearest gene (distance in bp)
chr7:27182493–27185137	7.03E−21	49	1.44E−20	2.40E−18	*AC004080.6*
chr7:27182493–27185137	7.03E−21	49	1.44E−20	2.40E−18	*HOXA*-*AS3*
chr7:27182493–27185137	7.03E−21	49	1.44E−20	2.40E−18	*HOXA3*
chr7:27182493–27185137	7.03E−21	49	1.44E−20	2.40E−18	*HOXA5*
chr7:27182493–27185137	7.03E−21	49	1.44E−20	2.40E−18	*HOXA6*
chr19:55972504–55973779	4.24E−13	11	1.37E−11	4.74E−09	*ISOC2*
chr6:28972937–28973829	8.00E−14	15	5.60E−10	2.77E−07	*ZNF311*
chr19:10735482–10736449	3.87E−08	11	2.56E−08	1.17E−05	*SLC44A2*
chr8:125462982–125463683	1.85E−08	7	2.40E−08	1.51E−05	*TRMT12*
chr12:54446019–54446577	4.07E−08	10	4.51E−08	3.57E−05	*HOXC4*
chr6:84418433–84419361	2.57E−07	13	1.70E−07	8.10E−05	*SNAP91*
chr16:89118603–89119710	3.98E−07	8	3.77E−07	1.50E−04	*AC135782.1*
chr17:7311282–7312082	3.96E−08	8	3.04E−07	1.68E−04	*NLGN2*
chr11:503807–504938	1.25E−06	8	7.47E−07	2.92E−04	*RNH1*
chr10:134600089–134601342	2.38E−09	21	1.19E−06	4.18E−04	*NKX6*-*2* (+ 1786)
chr3:5137549–5138171	5.51E−07	5	6.00E−07	4.26E−04	*AC090955.1* (− 3763)
chr21:47604052–47605175	8.51E−05	8	2.06E−06	8.10E−04	*AP001468.1*
chr21:47604052–47605175	8.51E−05	8	2.06E−06	8.10E−04	*SPATC1L*
chr1:6204000–6204225	5.34E−07	2	5.34E−07	1.05E−03	*CHD5*
chr5:178986131–178986907	1.42E−06	9	2.23E−06	1.27E−03	*RUFY1*
chr11:315751–316457	1.74E−06	6	2.03E−06	1.27E−03	*IFITM2* (+ 1185)
chr5:149669368–149669814	1.99E−06	6	1.99E−06	1.97E−03	*CAMK2A*
chr18:60051870–60052465	3.08E−06	5	3.28E−06	2.43E−03	*TNFRSF11A*
chr5:134879326–134880437	7.73E−05	6	6.71E−06	2.66E−03	*NEUROG1* (+ 8798)
chr18:7567426–7568892	3.67E−05	11	1.43E−05	4.29E−03	*PTPRM*
chr1:35586358–35586650	3.29E−06	5	3.29E−06	4.97E−03	*EFCAB14P1* (− 1265)
chr14:24422419–24423200	5.16E−06	8	9.16E−06	5.17E−03	*DHRS4*-*AS1*
chr14:24422419–24423200	5.16E−06	8	9.16E−06	5.17E−03	*DHRS4*
chr7:52341469–52342266	8.54E−06	5	9.85E−06	5.45E−03	*RF00322* (+ 5001)
chr22:34316316–34317025	1.66E−05	4	1.79E−05	1.12E−02	*LARGE*
chr6:30881112–30882261	1.12E−05	33	2.94E−05	1.12E−02	*GTF2H4*
chr6:30881112–30882261	1.12E−05	33	2.94E−05	1.12E−02	*VARS2*
chr21:46238781–46238958	5.09E−06	3	5.09E−06	1.26E−02	*SUMO3* (+ 264)
chr6:164393165–164393861	9.11E−06	7	2.30E−05	1.45E−02	*Z97205.2* (− 111890)
chr17:6899085–6899889	9.36E−07	14	3.81E−05	2.07E−02	*ALOX12*-*AS1*
chr17:6899085–6899889	9.36E−07	14	3.81E−05	2.07E−02	*ALOX12*
chr17:6899085–6899889	9.36E−07	14	3.81E−05	2.07E−02	*AC040977.2*
chr2:165811816–165812298	3.40E−06	6	2.72E−05	2.46E−02	*SLC38A11*
chr12:12848977–12849589	3.40E−05	10	3.55E−05	2.53E−02	*GPR19*
chr2:242823275–242824715	6.37E−04	8	8.72E−05	2.64E−02	*LINC01237*
chr7:27231204–27232151	6.53E−04	5	6.12E−05	2.82E−02	*AC004080.2*
chr17:4689284–4689894	4.00E−05	7	4.19E−05	2.99E−02	*VMO1*
chr11:73356744–73358108	1.59E−05	11	9.52E−05	3.04E−02	*PLEKHB1*
chr1:223566278–223567174	3.35E−05	10	7.17E−05	3.47E−02	*C1orf65*
chr12:49937852–49938019	1.35E−05	3	1.35E−05	3.50E−02	*KCNH3*
chr10:102880841–102881546	7.91E−06	7	6.20E−05	3.81E−02	*TLX1NB*
chr2:121776314–121776605	2.58E−05	3	2.58E−05	3.84E−02	*GLI2* (+ 26376)
chr3:182816873–182817627	7.39E−05	12	7.76E−05	4.45E−02	*MCCC1*
chr12:132293329–132293703	4.01E−05	5	4.01E−05	4.63E−02	*RNU6*-*1017P* (− 6479)
chr13:95363755–95364334	1.56E−03	4	6.32E−05	4.71E−02	*SOX21*
chr1:64668576–64669600	3.28E−04	12	1.17E−04	4.91E−02	*UBE2U*

*FDR* false discovery rate corrected p-value, *bp* base pairs

**Table 6:** DMRs associated to hand grip strength (FDR < 0.05) in the longitudinal EWAS (LSADT sample)

DMR position	Lowest p-value	Length	Region p-value	FDR p-value	Gene name/nearest gene (distance in bp)
chr13:97646341–97647223	5.71E−14	10	1.51E−12	7.55E−10	*OXGR1*
chr5:126852955–126853955	6.40E−14	16	1.54E−11	6.79E−09	*PRRC1*
chr19:48894694–48895031	3.46E−11	9	3.46E−11	4.53E−08	*KDELR1*
chr7:30544055–30545075	4.40E−13	14	3.51E−10	1.52E−07	*GGCT*
chr5:135414858–135416614	1.50E−07	20	8.51E−10	2.14E−07	*VTRNA2*-*1*
chr7:27141774–27143253	4.48E−10	19	3.16E−09	9.42E−07	*HOXA2*
chr1:16163479–16164123	6.11E−09	7	1.33E−08	9.15E−06	*FLJ37453*
chr22:32598479–32598571	4.18E−08	2	4.18E−08	2.01E−04	*RFPL2*
chr6:28956226–28956990	1.83E−08	22	8.13E−08	4.70E−05	*HCG16*
chr6:170054730–170055333	7.11E−08	3	9.50E−08	6.96E−05	*WDR27*
chr5:92956474–92957401	6.54E−08	11	1.73E−07	8.25E−05	*MIR2277*
chr5:92956474–92957401	6.54E−08	11	1.73E−07	8.25E−05	*FAM172A*
chr3:185911208–185912487	5.59E−06	9	2.06E−07	7.10E−05	*DGKG*
chr7:130130122–130132791	5.75E−06	60	4.19E−07	6.93E−05	*MEST*
chr10:135039948–135040251	5.60E−07	4	5.60E−07	8.16E−04	*KNDC1* (+ 335)
chr17:35423405–35424060	4.76E−07	6	9.87E−07	6.65E−04	*AATF* (+ 9889)
chr14:101290717–101293451	3.36E−05	30	1.02E−06	1.65E−04	*MEG3*
chr11:128693567–128694680	1.14E−08	10	1.52E−06	6.03E−04	*FLI1* (+ 11518)
chr18:77280171–77280587	1.92E−06	4	1.92E−06	2.04E−03	*NFATC1*
chr8:1094484–1094905	2.02E−06	6	2.02E−06	2.11E−03	*ERICH1*-*AS1* (+ 7128)
chr5:162864268–162865237	1.03E−06	13	2.14E−06	9.77E−04	*CCNG1* (− 307)
chr5:162864268–162865237	1.03E−06	13	2.14E−06	9.77E−04	*CCNG1*
chr11:15095017–15095979	2.35E−06	10	2.25E−06	1.03E−04	*CALCB*
chr5:180045597–180046053	2.15E−06	3	2.74E−06	2.65E−03	*FLT4*
chr4:39448160–39449131	2.45E−06	7	3.40E−06	1.55E−03	*KLB*
chr5:134362967–134364718	1.05E−05	11	5.66E−06	1.43E−03	*PITX1*
chr12:247963–249287	4.05E−05	6	5.71E−06	1.90E−03	*IQSEC3*
chr12:247963–249287	4.05E−05	6	5.71E−06	1.90E−03	*AC026369.1*
chr6:170589483–170589898	7.25E−06	4	7.25E−06	7.69E−03	*LINC01624* (+ 923)
chr8:61626185–61627282	5.49E−09	6	7.69E−06	3.09E−03	*CHD7*
chr1:203320190–203320542	1.41E−05	6	1.41E−05	1.75E−02	*FMOD*
chr20:57463265–57465140	1.36E−05	41	1.44E−05	3.38E−03	*AL121917.1*
chr20:57463265–57465140	1.36E−05	41	1.44E−05	3.38E−03	*GNAS*
chr10:131263962–131265797	6.68E−06	27	1.64E−05	3.95E−03	*MGMT*
chr13:112209007–112209222	1.67E−05	3	1.67E−05	3.37E−02	*AL359649.1* (− 31541)
chr13:100217962–100219014	8.56E−05	6	1.70E−05	7.10E−03	*TM9SF2* (+ 2754)
chr14:23623480–23624378	1.44E−05	8	1.80E−05	8.79E−03	*SLC7A8*
chr18:77905119–77905948	1.37E−05	10	1.88E−05	9.95E−03	*ADNP2*
chr18:77905119–77905948	1.37E−05	10	1.88E−05	9.95E−03	*PARD6G*-*AS1*
chr3:67704629–67705286	9.14E−06	7	2.26E−05	1.51E−02	*SUCLG2*-*AS1*
chr3:67704629–67705286	9.14E−06	7	2.26E−05	1.51E−02	*SUCLG2*
chr6:33872206–33872862	5.35E−05	7	2.98E−05	1.98E−02	*AL138889.1*
chr7:157981849–157982590	2.24E−05	8	3.21E−05	1.89E−02	*PTPRN2*
chr2:61371880–61372317	3.56E−05	9	3.56E−05	3.53E−02	*AC016747.1*
chr2:61371880–61372317	3.56E−05	9	3.56E−05	3.53E−02	*KIAA1841*
chr2:61371880–61372317	3.56E−05	9	3.56E−05	3.53E−02	*C2orf74*
chr22:38092643–38093208	2.42E−05	11	3.80E−05	2.93E−02	*NOL12*
chr22:38092643–38093208	2.42E−05	11	3.80E−05	2.93E−02	*TRIOBP*
chr2:177013630–177015126	5.13E−05	15	3.82E−05	1.12E−02	*MIR10B*
chr2:177013630–177015126	5.13E−05	15	3.82E−05	1.12E−02	*HOXD3*
chr11:2019129–2020561	8.85E−05	38	3.93E−05	1.20E−02	*H19*
chr4:10020360–10021356	1.80E−04	6	4.06E−05	1.78E−02	*SLC2A9*
chr6:109611667–109612230	4.59E−05	4	5.66E−05	4.35E−02	*PTCHD3P3*
chr14:103366987–103367859	9.99E−05	6	5.82E−05	2.91E−02	*TRAF3*
chr8:1365049–1365907	4.61E−05	6	6.88E−05	3.48E−02	*AF067845.1* (+ 47908)
chr22:31317764–31318547	1.47E−05	12	6.94E−05	3.84E−02	*MORC2*-*AS1*
chr6:32144667–32146780	2.34E−05	39	7.14E−05	1.48E−02	*AGPAT1*
chr6:32144667–32146780	2.34E−05	39	7.14E−05	1.48E−02	*RNF5*
chr7:90895894–90896702	6.45E−05	4	7.36E−05	3.95E−02	*FZD1*
chr11:6290951–6292897	1.12E−04	13	8.42E−05	1.89E−02	*CCKBR*
chr1:206858537–206859687	3.68E−05	9	9.04E−05	3.42E−02	*MAPKAPK2*
chr16:68482144–68483195	5.76E−05	12	1.05E−04	4.31E−02	*SMPD3*
chr12:19592184–19593685	5.58E−04	10	1.26E−04	3.63E−02	*AEBP2*

*FDR* false discovery rate corrected p-value, *bp* base pairs

Moreover, a number of genes were observed in more than one comb-p analysis (Online Resource 1, Supplementary Table 8): the major histocompatibility complex, class II, DP alpha 1 (*HLA*-*DPA1*), *AP001468.1* (RNA antisense gene), coiled-coil domain containing 185 (*C1orf65*), long intergenic non-protein coding RNA 299 (*LINC00299*), zinc finger protein 311 (*ZNF311*) and spermatogenesis and centriole associated 1 like (*SPATC1L*) genes were seen in the individual level EWAS and the twin pair level EWAS, while the uncharacterized LOC729614 (*FLJ37453*), mesoderm specific transcript (*MEST*) and long intergenic non-protein coding RNA 1624 (*LINC01624*) genes were observed in the longitudinal EWAS, as well as in a cross sectional EWAS. The latter finding might indicate that *FLJ37453*, *MEST* and *LINC01624* could be relevant for both the level of hand grip strength at one point in time and the spanning over several time points. The biological functions of *FLJ37453* and *LINC01624* are largely unknown, while *MEST* is known to encode an alpha/beta hydrolase protein. Mutations in *MEST* cause among others the Russell–Silver syndrome (omim.org/entry/180860), characteristics including short stature.

Major histocompatibility complex genes were also revealed in the gene sets passing correction for multiple testing in the genomic regions enrichment analysis (GREA) (Table 7): the major histocompatibility complex, class I, C (*HLA*-*C*), major histocompatibility complex, class I, B (*HLA*-*B*), major histocompatibility complex, class II, DM alpha (*HLA*-*DMA*) and major histocompatibility complex, class II, DP beta 1 (*HLA*-*DPB1*) genes did, together with the (non-muscular) myosin heavy chain 10 (*MYH10*), endoplasmic reticulum aminopeptidase 1 (*ERAP1*), and interferon regulatory factor 8 (*IRF8*) genes, constitute the 7 pathways related to the immune system or (auto)immune diseases. Some of these genes have been reported to be mutated in diseases related to the immune system, e.g. *HLA*-*DMA*: rheumatoid arthritis and lupus, *HLA*-*C*: psoriasis and HIV, *HLA*-*B*: ankylosing spondylitis (arthritis of the spine) and *IRF8* and *HLA*-*DPB1*: immune deficiencies (www.genecards.org). Moreover, when considering all nominally significant findings from the GREA (Online Resource 1, Supplementary Table 8), 31 out of 80 unique gene sets were related to immune function. These findings point to immune function as important for hand grip strength, which might not be surprising considering the well-known role of the immune system in muscle growth and regeneration (Tidball 2017), a process which declines during aging, contributing to the occurrence of sarcopenia (Domingues-Faria et al. 2016).

**Table 7:** Pathways passing correction for multiple testing (FDR < 0.05) in GREAT genomics regions enrichment analysis (GREA) of comb-p identified DMRs (individual level EWAS)

Overall level of functional class	Name (ID)	P-value	FDR p-value	Fold enrichment	Expected	Observed gene hits	Total genes	Gene set coverage	Term gene coverage
Human diseases; Cardiovascular diseases	Viral myocarditis (KEGG hsa05416)	1.80E−05	0.024	15.42	0.32	*HLA*-*DMA, HLA*-*DPB1, HLA*-*C, HLA*-*B, MYH10*	68	0.058	0.074
Human diseases; Immune diseases	Allograft rejection (KEGG hsa05330)	2.25E−05	0.015	23.97	0.17	*HLA*-*DMA, HLA*-*DPB1, HLA*-*C, HLA*-*B*	35	0.047	0.114
Human diseases; Immune diseases	Graft-versus-host disease (KEGG hsa05332)	2.82E−05	0.012	22.68	0.18	*HLA*-*DMA, HLA*-*DPB1, HLA*-*C, HLA*-*B*	37	0.047	0.108
Human diseases; Endocrine and metabolic diseases	Type I diabetes mellitus (KEGG hsa04940)	4.26E−05	0.014	20.47	0.20	*HLA*-*DMA, HLA*-*DPB1, HLA*-*C, HLA*-*B*	41	0.047	0.098
Human diseases; Immune diseases	Autoimmune thyroid disease (KEGG hsa05320)	9.38E−05	0.025	16.78	0.24	*HLA*-*DMA, HLA*-*DPB1, HLA*-*C, HLA*-*B*	50	0.047	0.080
Immune system	Genes involved in Antigen Presentation: Folding, assembly and peptide loading of class I MHC (Reactome R-HSA-983170)	1.12E−04	0.025	31.47	0.10	*ERAP*-*1, HLA*-*C, HLA*-*B*	20	0.035	0.150
Immune system	Genes involved in Interferon gamma signaling (Reactome R-HSA-877300)	1.91E−04	0.036	13.99	0.29	*HLA*-*DPB1, HLA*-*C, HLA*-*B, IRF8*	60	0.047	0.067
NA	Validated targets of C-MYC transcriptional repression (PID_MYC_REPRESS_PATHWAY)	2.31E−04	0.038	13.32	0.30	*CEBPD, ID2, IRF8, BRCA1*	63	0.047	0.064

*FDR* false discovery rate corrected p value, *Fold enrichment* fold enrichment of number of genomic regions in the test set with the annotation, *Expected* expected number of genomic regions in the test set with the annotation, *Observed gene hits* actual number of genomic regions in the test set with the annotation, *Total Genes* number of genes in the genome with the annotation, *Set Coverage* the fraction of all genes in the test set with the annotation, *Term Coverage* fraction of all genes with the annotation that are tagged by the test set

The last gene set found in the GREA related to negative regulation of cell differentiation and was composed of the *IRF8*, the CCAAT enhancer binding protein delta (*CEBPD*), inhibitor of DNA binding 2 (*ID2*) and BRCA1 DNA repair associated (*BRCA1*) genes. The *MYC10*, *ID2* and *BRCA1* genes are involved in cancer, while *CEBPD* is involved in juvenile diseases of the central nervous system, as well as in regulation of genes involved in immune and inflammatory responses (www.genecards.org). Furthermore, as mentioned in the introduction, an interaction partner of *CEBPD*, *CEBPB*, has been implicated in muscle cell differentiation and muscle repair (Matteini et al. 2016), and was reported in the TWAS by Harries et al. (2012). Also, *ID2* has been related to muscle cell differentiation (www.genecards.org), indicating a role of these two genes in muscle biology, potentially mediated via repression of cell differentiation or immune function.

Finally, in the GREA the individual level EWAS displayed significant findings (Table 7), while the twin pair level EWAS and the longitudinal EWAS did not. One reason could be that the latter holds lower power, as it only included the elderly part of the study population. Moreover, the twin pair EWAS controls for genetic variation by analyzing intra-pair differences by linear regression, while the individual level EWAS accommodate the intra-pair correlation by inclusion of twin pairing as a random factor, which relies on the fulfillment of the assumptions of the model. Hence, it is possible that the individual level EWAS results are, least in part, influenced by genetic variations left unaccounted for.

A possible limitation of the present study is the use of DNA methylation data from whole blood rather than a tissue more intuitively relevant for a muscle related trait. It is well known that epigenetics is important for differentiation of and aging in muscle cells (Carrió and Suelves 2015; Zykovich et al. 2014), and that, although there is some overlap, differences in the epigenetic signature of muscle tissue and blood cells have been reported (Slieker et al. 2013). The findings by Livshits et al. (2016) on muscle mass do, however, indicate that the use of blood samples for epigenetic studies is a feasible approach for identification of muscle-related epigenetic changes, although obviously restricted to changes that are mirrored in blood. This line of thought is supported by the blood-based gene expression study performed by Pilling et al. (2016) on hand grip strength, which found several genes previously reported to be associated to muscle function (Pilling et al. 2016). In any case, to decipher the muscle cell specific epigenetic signatures related to muscle strength, future studies on the less accessible muscle cell derived DNA methylation data and hand grip strength are highly relevant. Furthermore, as the age-related decline in muscle strength is a multifactorial and complex process affected by for instance cognitive, neural and hormonal processes (Manini and Clark 2012; Tieland et al. 2018), we cannot exclude that other physiological aging-related phenomena are reflected in the blood samples under study here, and that the results obtained might reflect such phenomena. Specifically, we can not exclude that the findings might, at least in part, reflect frailty, especially in the elderly part of the study population, as decreased hand grip strength is one component of the frailty phenotype (e.g. Li et al. 2019). Most importantly, this point is supported by the fact that the most significant biological pathways found in this study are highly implicated in immune function. In the literature, the strong connection between immune function and physical frailty has been extensively studied and discussed (Lu et al. 2016; Fuentes et al. 2017). In this sense, findings from this exploratory study might provide potential references for exploring the epigenetics of age-related loss of physical capacity.

In conclusion, in this exploratory study we identified several epigenetic markers of potential importance to physical functioning, as reflected by hand grip strength, using both cross-sectional and longitudinal data from twins. Furthermore, investigation of differentially methylated regions identified significant genomic regions and pathways related to the immune system and autoimmune diseases. However, additional studies are needed to further clarify the potential importance of these markers and regions. Such clarification could elucidate the mechanisms underlining maintenance and loss of muscle strength and age-related loss of physical capacity.

## Electronic supplementary material

Below is the link to the electronic supplementary material.

Supplementary material 1 (XLSX 95703 kb)

## Data Availability

According to the Danish and EU legislations, transfer and sharing of individual-level data require prior approval from the Danish Data Protection Agency and require that data sharing requests are dealt with on a case-by-case basis. For this reason, the raw data cannot be deposited in a public database. However, we welcome any enquiries regarding collaboration and individual requests for data sharing.

## Abbreviations

EWAS: Epigenome-wide association study
GWAS: Genome-wide association study
TWAS: Transcriptome-wide association study
MADT: The Study of Middle Aged Danish Twins
LSADT: The Longitudinal Study of Aging Danish Twins
BWD: The Study of Birth Weight Discordant Twins
PCA: Principle component analysis
DMRs: Differentially methylated regions
FDR: False discovery rate
TSS: Transcription start site
KEGG: The Kyoto Encyclopedia of Genes and Genomes database
WebGestalt: The WEB-based GEne SeT AnaLysis Toolkit
MSigDB: Molecular Signatures Database
GREAT: Genomic regions enrichment annotation tool
ORA: Overrepresentation enrichment analysis
GREA: Genomic regions enrichment analysis
